# Twenty Metabolic Genes Based Signature Predicts Survival of Glioma Patients

**DOI:** 10.7150/jca.30923

**Published:** 2020-01-01

**Authors:** Wenfang Xu, Zhenhao Liu, He Ren, Xueqing Peng, Aoshen Wu, Duan Ma, Gang Liu, Lei Liu

**Affiliations:** Department of Biochemistry and Molecular Biology, School of Basic Medical Sciences and Institutes of Biomedical Sciences, Fudan University, 200032, Shanghai, P.R.China.

**Keywords:** Glioma, Marker, Risk Score, Random Forest Variable Hunting, Metabolic Genes

## Abstract

**Background:** Glioma, caused by carcinogenesis of brain and spinal glial cells, is the most common primary malignant brain tumor. To find the important indicator for glioma prognosis is still a challenge and the metabolic alteration of glioma has been frequently reported recently.

**Methods:** In our current work, a risk score model based on the expression of twenty metabolic genes was developed using the metabolic gene expressions in The Cancer Genome Atlas (TCGA) dataset, the methods of which included the cox multivariate regression and the random forest variable hunting, a kind of machine learning algorithm, and the risk score generated from this model is used to make predictions in the survival of glioma patients in the training dataset. Subsequently, the result was further verified in other three verification sets (GSE4271, GSE4412 and GSE16011). Risk score related pathways collected in the Kyoto Encyclopedia of Genes and Genomes (KEGG) database were identified using Gene Set Enrichment Analysis (GSEA).

**Results:** The risk score generated from our model makes good predictions in the survival of glioma patients in the training dataset and other three verification sets. By assessing the relationships between clinical indicators and the risk score, we found that the risk score was an independent and significant indicator for the prognosis of glioma patients. Simultaneously, we conducted a survival analysis of the patients who received chemotherapy and who did not, finding that the risk score was equally valid in both cases. And signaling pathways related to the genesis and development of multiple cancers were also identified.

**Conclusions:** In summary, our risk score model is predictive for 967 glioma patients' survival from four independent datasets, and the risk score is a meaningful and independent parameter of the clinicopathological information.

## Introduction

Gliomas account for 70% of brain cancers, of which, 101,600 new cases and 61,000 related deaths have been reported, according to the latest statistic reports in China, 2015 1. The median survival time of glioma patients is only about 12 months 2, and the five-year survival rate of glioma patients is less than 3% 3. Clinically, World Health Organization (WHO) classifies gliomas into different grades according to the pathological observations 4. And yet, the implementation of the staging system is not predictive of prognosis. Therefore, bio-molecular markers are needed to predict glioma patients' survival.

Over the past decades, bio-molecular markers for glioma prognosis have been extensively reported 5-7. Among these biomarkers, metabolic genes are especially important. For example, IDH1.R132H and IDH2.R172H mutations have been frequently reported in glioma cases 8, and patients harboring IDH1 and IDH2 mutations have a different metabolic pattern^9^ and usually have a better prognosis compared to patients with IDH1/2 wild type 10, 11. Methylation of O-6-methylguanine-DNA methyltransferase (MGMT) is another metabolic biomarker for prognosis, and is clinically relevant to the efficacy of the treatment 12, 13. In addition, the metabolic status of glioma stem cells is distinct from that of the adjacent normal tissues 14. However, single bio-molecular marker usually fails to predict glioma patients' survival in consideration of the heterogeneity of cancer, and classification based on transcriptome level contains a large amount of redundant information. On the contrary, the robustness of models based on multiple molecular biomarkers have been proved across datasets and applied to other cancers 15-17.

In this vein, we selected genes related to survival based on metabolic gene expressions, and built a prognostic model using Random Forest machine learning algorithm and Cox regression. The model accurately categorized the patients into good prognosis and poor prognosis groups, and the result was verified in other three validation sets. The evaluation of correlations between clinical indicators and the risk score shows that the independence of risk score compared to other clinical features and the score outperforms other clinical observations in predicting the patients' survival. Meanwhile, the score is valid for patients who received chemotherapy and who didn't. KEGG pathway analysis shows that multiple pathways associated with cancer changed significantly between the high-risk group and the low-risk group, including apoptosis and JAK-STAT signaling pathways.

## Methods

### Data pre-processing

The raw gene expression data and clinical data were obtained from University of California Santa Cruz (UCSC) Xena and Gene Expression Omnibus (GEO) databases. Background proofing and Robust Multichip Average standardization were implementted among samples in each batch. Subsequently, probes were corresponded to Entrez gene names by referring to the annotation files provided by manufacture in all datasets and platforms. For genes matching more than one probes, average values were regarded as the relative expressions. Genes involved in metabolism were extracted from the previous report 18, and these genes were retained for further analysis. The R Language was used for the statistical analysis of clinical information.

### Marker selection and model establishment

Cox univariate regression was used to assess the correlation between the overall survival and the expression level of each metabolic gene with R, and significant genes (p<0.01) were retained for further analysis. Subsequently, random forest variable hunting was implemented to select the most important genes to establish the predicting model 19, 20. The number of iterations and repeats are both 100. Based on the expression of screened genes, the risk score model was developed through Cox multivariate model as the following formula:


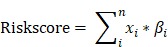


Where x*_i_* indicates the expression of gene *i*, meanwhile β*_i_* means the coefficient of gene *i* generated from the Cox multivariate regression.

### Statistical applications and pathway analysis

R function survival::coxph was used for the Cox multivariate and univariate regression analysis, and R function randomForestSRC::var.select was used for random forest survival analysis 21 using the following parameters: 100 repeats, 100 iterations. The risk score difference between categories divided by clinical indicators was calculated based on Student's -test. R package “rms” was used for nomogram calculation and visualization. GSEA 22 was implemented using a java software developed and the default parameters were used by comparing the high-risk (higher than median) and low-risk group (lower than median).

## Results

### Marker selection and model establishment

**Figure 1:**
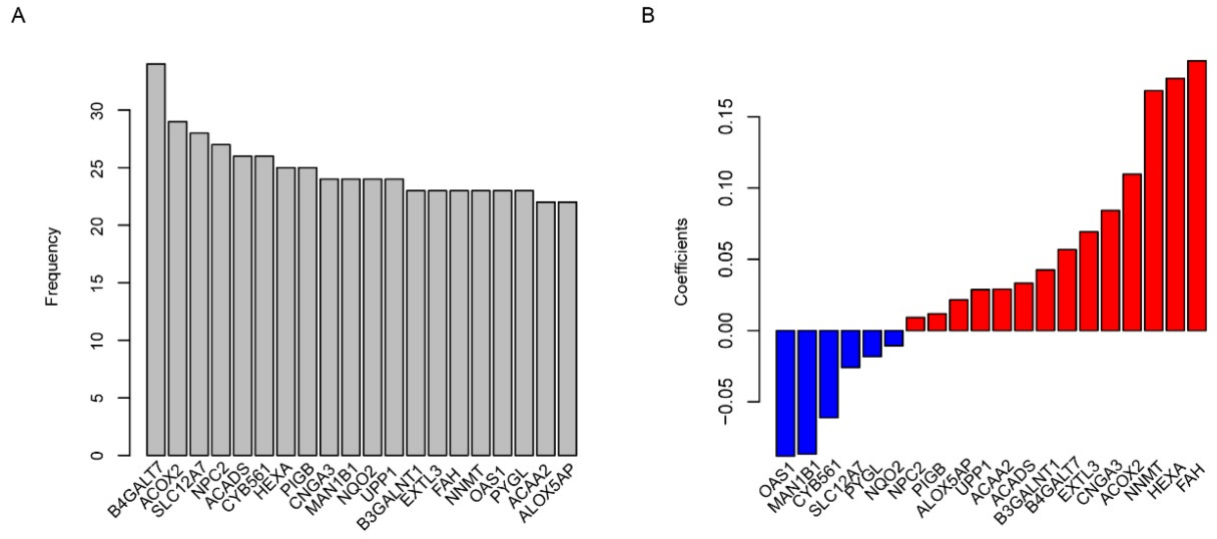
Risk score model development. The frequency of genes presented in random forest variable hunting (A) and the coefficient for each gene (B).

Metabolic genes and related metabolic pathways were reserved for further analysis and the other genes were excluded. In order to find out the survival associated metabolic genes, we used Cox univariate regression to analyze the relationship between overall survival time and gene expression evaluated by microarray (U133A) in TCGA dataset (N=529, median survival: 12 months). 101 genes were identified (p<0.01) and regarded as survival associated genes. We used Random Forest machine learning algorithm to remove redundant information from the selected genes, and twenty genes were filtered for further studies (Fig. 1A, Table 1). The functions of these 20 genes were described in detail in Table S1. The coefficients of OAS1, MAN1B1, CYB561, SLC12A7, PYGL and NQO2 were negative numbers (Fig. 1B), suggesting that the high expression of these genes was significantly associated with longer overall survival time.

### Prediction of risk score model

**Fig 2:**
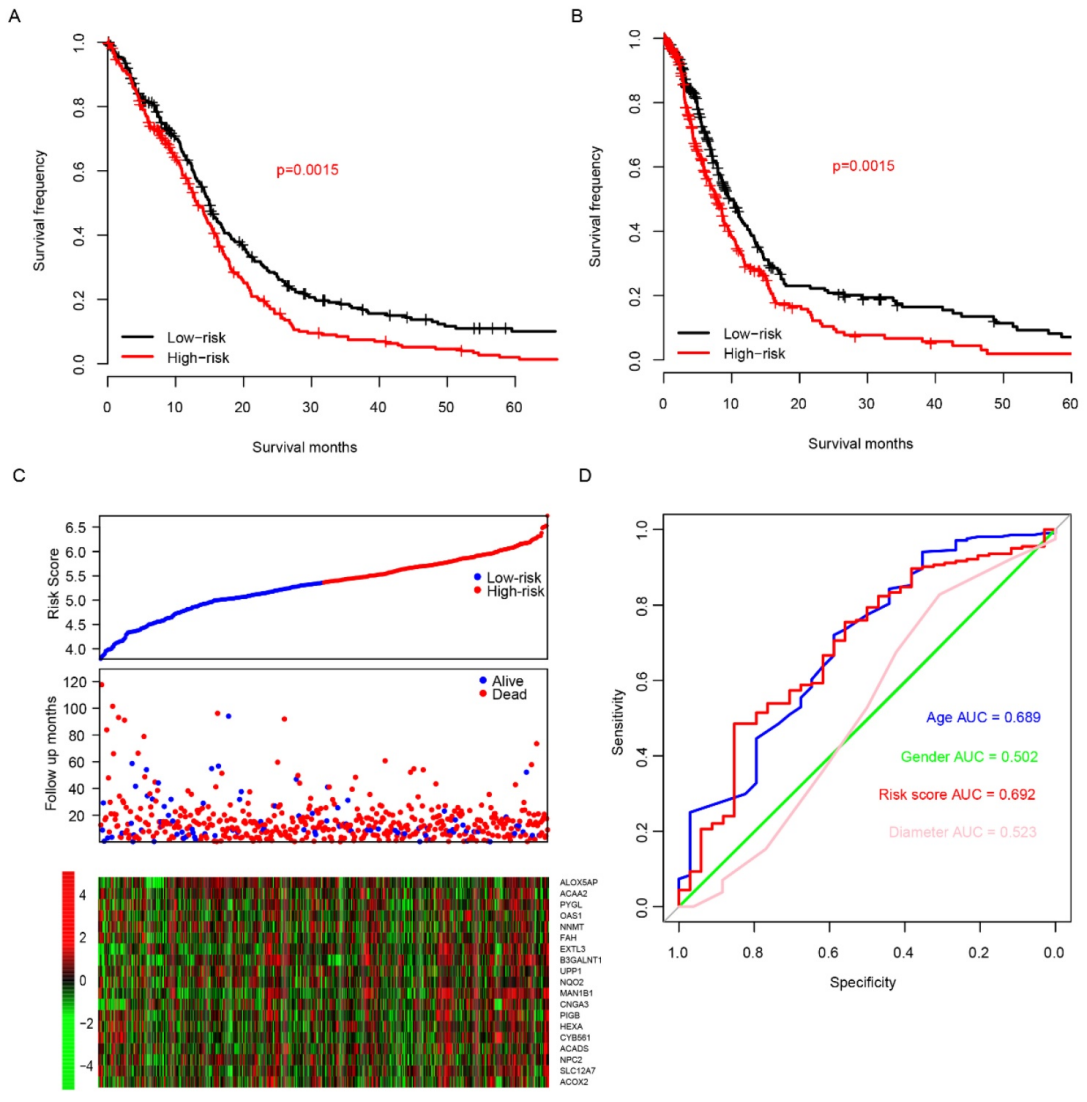
Risk score in predicting survival. The high-risk group has a significantly longer overall survival (OS) time than low risk group (A), and it is also similar to progression free survival (PFS, B). Detailed survival information of samples, risk score and gene expression (each point represents a sample, C) and one-year survival ROC were also plotted (D).

Using the TCGA dataset as the training set, the predictive value of the risk score model was estimated. The glioma patients were grouped into two groups by the median value of the risk score. The overall survival time is 14.9 (95% CI: 13.5-16.7) months in low-risk group, which was remarkably longer (p=0.0015) than 13.1 months (95% CI: 12.0-15.1), the median survival time of high-risk group (Fig. 2A). As shown in Fig. 2B, the comparison of progression-free survival (PFS) between the two groups also showed the same trend (p=0.0015). The sooner the patients' events occurred as the risk score increased, and the risk score is positively correlated with the expression of oncogenes and negatively with suppressor genes in twenty candidate genes (Fig. 2C). One-year survival area under receiving operating characteristic (AUROC) curve was plotted, and the AUROC for age, gender, risk score and primary tumor diameter were 0.689, 0.502, 0,692 and 0.523 (Fig. 2D), indicating that the risk score is a strong prognostic indicator for glioma patients.

### Validation of risk score

**Fig 3:**
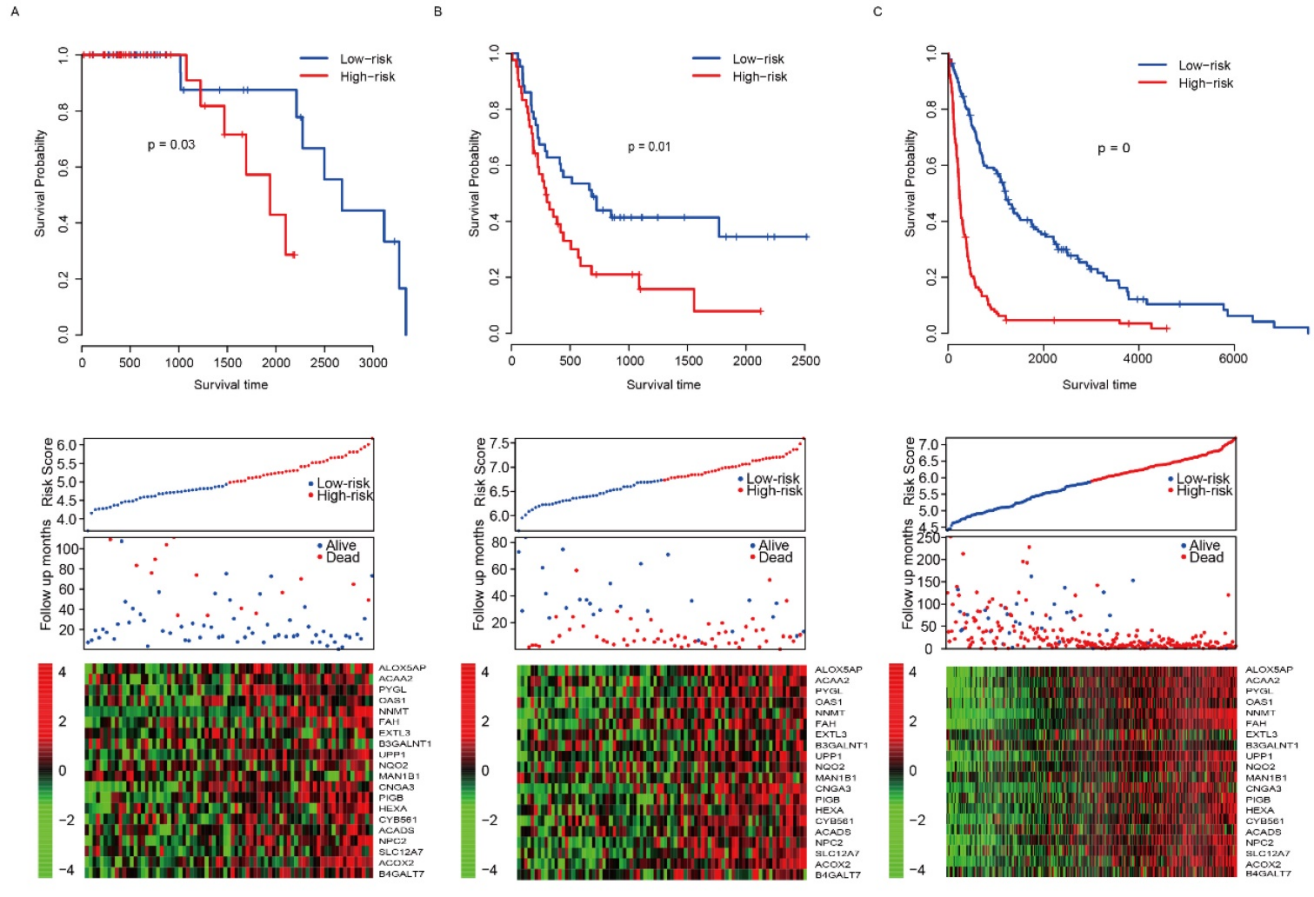
Risk score performance validation. The performance of risk in predicting survival was validated in GSE4271 (A, top panel), GSE4412 (B, top panel) and GSE16011 (C, top panel) datasets. The detailed survival information and gene expression of the three dataset (A-C, middle and bottom panel) also resembles the profile of training dataset (TCGA).

The good predictive effect of the risk score in the training set might be due to the overfitting between the data and the model. For further validation, we used other three independent datasets, GSE4271 23, GSE4412^24^ and GSE16011^25^, to evaluate the prognostic robustness of the risk score. Using the median risk score value, we categorized the samples into two groups in each dataset, and the survival differences were analyzed. In group whose patients with low risk score, the patients' survival time is obviously longer in all of the validation sets (p=0.03, 0.01 and 0 for GSE4271, GSE4412 and GSE16011, respectively, Fig. 3A-C, top panel). Same as the training set, we found that the patients were dead sooner as the risk score increased in each verification dataset (Fig. 3A-C, middle panel). Furthermore, the relationship between the expression of the 20 candidate genes and the risk score was also similar to that of the training set (Fig. 3A-C, bottom panel). These results suggest that this model has considerable robustness in predicting the prognosis of glioma patients.

### Risk score is independent of other clinical indicators

**Fig 4:**
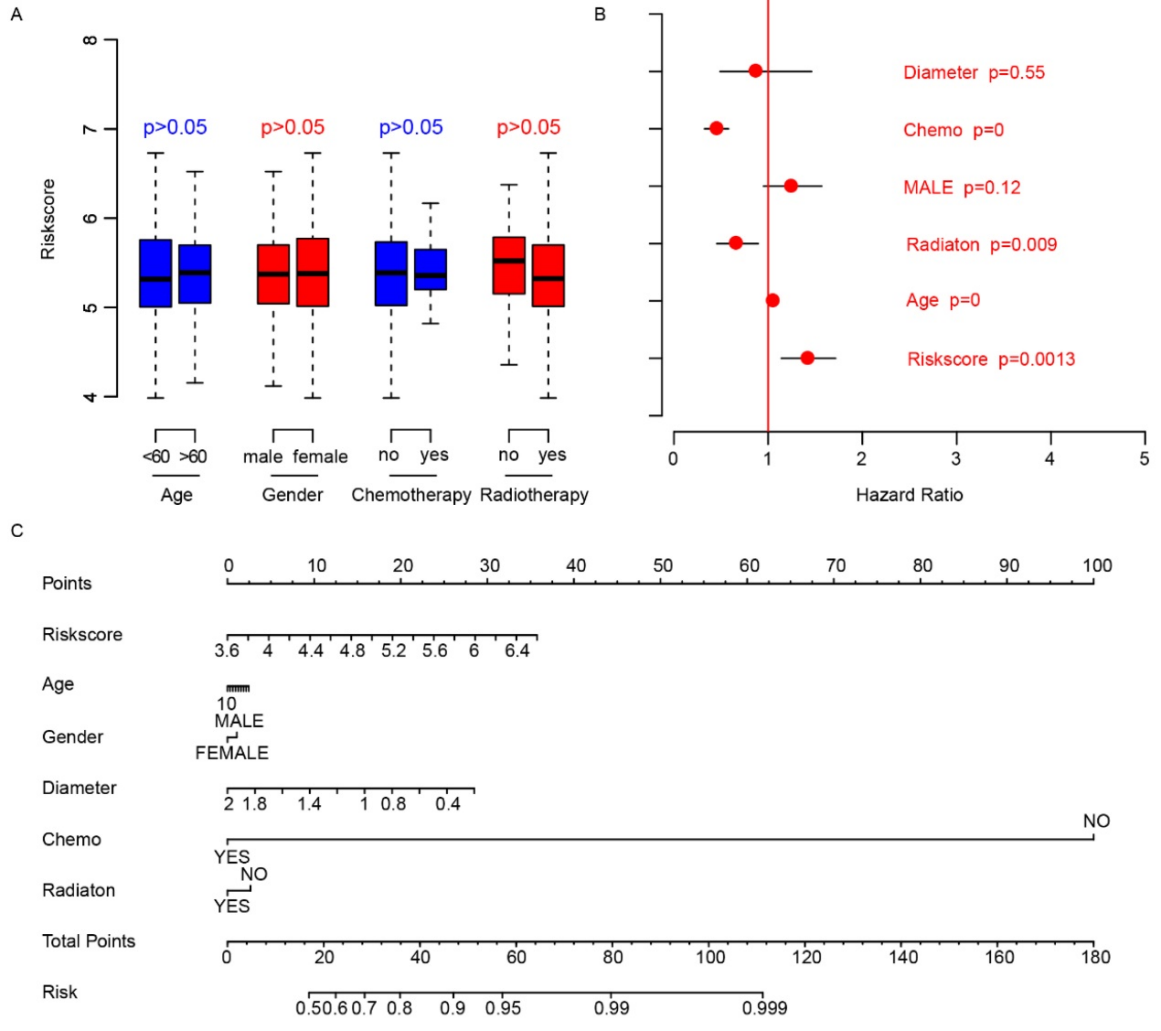
Clinical observations and risk score. The distributions of risk score in age (<60, >60), gender (male, female), chemotherapy (no-not received, yes-received) and radiotherapy (no-not received, yes-received) are shown (A), and the risk score is an important indicator for survival (B) according to Cox multivariate regression. The nomogram was plotted to facilitate utilization of risk score (C).

We did a survey of the clinical information of patients in each dataset (Table 2). The relationship between the risk score and other clinical indicators was explored. Firstly, we grouped the patients by other clinical indicators including age, gender, chemotherapy, and radiotherapy, respectively. Then we analyzed whether there were differences in risk scores within the categories divided by each clinical indexes. The results showed that these indicators were independent of the risk score (Fig. 4A). Thereafter, we used Cox multivariate regression to analyze the significance of age, gender, diameter, chemotherapy, radiation and risk score in the prediction of clinical outcome. The result indicated that the risk score was a valuable prognostic predictor (Fig. 4B). A nomogram was plotted using the aforementioned clinical information to promote the application of risk score (Fig. 4C). The above results showed the importance and independence of the risk score as a candidate prognostic indicator.

### Risk score and chemotherapy

**Fig 5:**
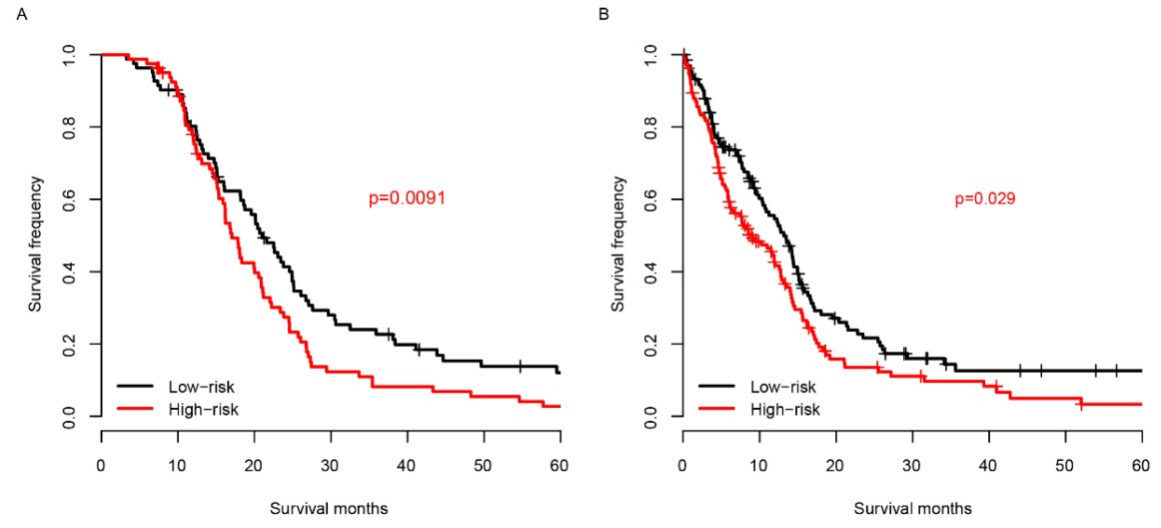
Risk score and chemotherapy. The risk score successfully predicts the survival of patients who received chemotherapy (A) and who did not (B).

Chemotherapy is one of the most essential auxiliary treatments for glioma. So, the risk score performance was investigated in patients who received chemotherapy and who did not. The patients receiving chemotherapy were partitioned into high-risk group and low-risk group based on the median risk score of TCGA samples. As shown in Fig. 5A, among the patients who received chemotherapy, the prognosis of the low-risk group was significantly better than that of the high-risk group. The survival distribution of patients without chemotherapy is similar to that of patients with chemotherapy (Fig. 5B). These results reveal that the score is effective for patients who received chemotherapy and who did not.

### Risk score related pathway analysis

**Fig 6:**
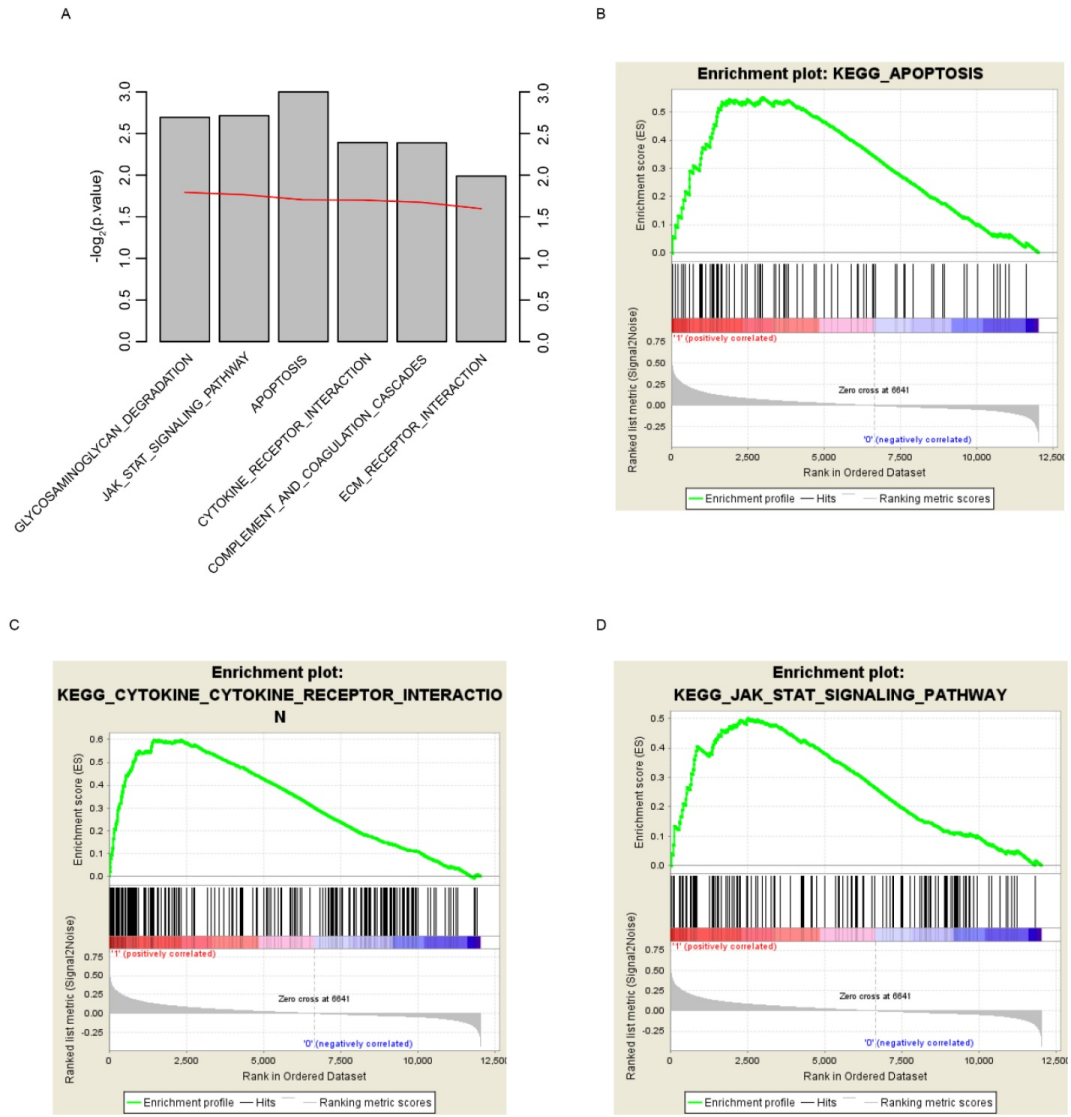
KEGG pathways associated with risk score. GSEA based on the expression of TCGA dataset revealed significant (p<0.01) pathways associated with risk score (A), including apoptosis (B), cytokine-cytokine receptor interaction (C) and JAK-STAT signaling pathway (D). For each gene set, vertical bars along the x-axis of the GSEA plot represent the positions of genes within the ranked list (i.e. their fold change). Negative GSEA enrichment score curve indicates anti-enrichment (down-regulation), and positive curve denotes enrichment (up-regulation) (B, C, D).

In order to find out the reason why risk score could predict glioma patients' survival, here we grouped the samples according to the median risk score, which are high and low risk groups. GSEA was implemented to investigate the altered pathways between high and low risk groups. Various cancer associated signaling pathways, including glycosaminoglycan degradation, JAK-STAT signaling pathway, apoptosis, cytokine receptor interaction, complement and coagulation cascades, and ECM receptor interaction, were significantly enriched (Fig. 6A, p<0.05). Among these pathways, apoptosis, cytokine receptor interaction and JAK-STAT signaling pathway were shown (Fig. 6B-D). According to these results we draw the conclusion that the survival of glioma patients can be accurately predicted by the risk score, perhaps because the score can reflect the multi-level status of glioblastoma.

## Discussion

Metabolism alterations of glioma have been frequently reported in the past years. The major mutation in glioma is IDH1/IDH2, which is a key metabolic gene in the oxidative decarboxylation of isocitrate in tricarboxylic acid (TCA) cycle, converting isocitrate to ɑ-ketoglutarate as reducing NADP+ to NADPH^26^. Mutations of IDH1/2 convert isocitrate to a toxic metabolite 2-hydroxyglutarate instead^27^. In addition to mutations in IDH1/2, the expressions of other metabolic enzymes also play an important role in carcinogenesis^28^ and cancer development^29^. However, single biomarker is not robust in predicting the survival. For example, none of the genes that were significantly associated with survival could be detected in all the datasets we used in this study. Recent studies highlight the robustness of multiple genes in predicting survival of cancer patients^30^-^32^. In our current work, by utilizing the expressions of metabolic genes, we developed a model to predict the survival of glioma patients, and validated its effect in three independent datasets. The clinical significance of risk score was evaluated and associated KEGG pathways were identified.

It is noted that these 20 genes are involved in different metabolic categories. For example, ACADS and ACOX2 are involved in fatty acid metabolism^33^, ^34^; B3GALNT1, B4GALT7, HEXA and MAN1B1 are in glycan synthesis and metabolism^35^-^38^; other genes are categorized into ion transport (CNGA3 and SLC12A7)^39^, ^40^, NAD metabolism (NNMT, NQO2)^41^, ^42^, and redox and tyrosine metabolism (FAH)^43^, indicating that aberrant gene expressions in multiple metabolic pathways affect the prognosis of glioma. Among these metabolic genes, most have not been reported to be associated with prognosis of glioma patients, and only ACOX2 is involved in the prognosis of breast cancer^44^. Although few of these genes we screened for cancer prognosis are reported, to some extent they can reflect the status of the cancer-driven genes related to their upstream and downstream. These genes we screened are enriched in multiple cancer-related pathways. According to these results, we draw the conclusion that the survival of glioma patients can be accurately predicted by the risk score, perhaps because the score can reflect the multi-level status of glioblastoma. However, it is unclear how these genes play their own role in the mechanism and so exploring the impact of metabolic enzymes on survival requires more investigations.

Limitations of this study exist. Firstly, it is a retrospective study. Thus, information including time to recurrence, treatment records and detailed pathological stage was unavailable. Secondly, although the model was validated across cohorts, it still need more samples to further confirm before clinical utilization. Last but not least, the genes in the model was optimized, but still it is a locally optimal solution instead a global optimal solution. One of the evidence is that the p values of these 20 genes in multivariate regression was mostly >0.05.

## Conclusions

Our results show that the risk score based on 20 metabolic genes' expression is effective for predicting the survival of glioma patients. Meanwhile, the risk score is independent from other clinical indicators. Moreover, the score can reflect the multi-level status of glioblastoma. Our research might provide a new approach to the prognosis of glioma patients and motivate basic medical research on the prognosis of glioma.

## Supplementary Material

Supplementary figures and tables.

## Figures and Tables

**Table 1:** Hazard ratio (HR), 95% confidence interval (CI), p values of candidate genes according to Cox univariate and multivariate regression

Gene		Univariate			Multivariate		
Entez ID	Gene symbol	HR	95% CI	pvalue	HR	95% CI	pvalue
241	ALOX5AP	1.1	1-1.2	0.00484	1.06	0.96-1.17	0.2784
10449	ACAA2	1.2	1.1-1.3	0.00329	1.12	0.97-1.29	0.12932
5836	PYGL	1.2	1.1-1.3	0.00093	0.97	0.85-1.11	0.70363
4938	OAS1	1.1	1-1.2	0.0073	1.01	0.92-1.1	0.84306
4837	NNMT	1.1	1-1.1	0.00011	1.03	0.98-1.1	0.26509
2184	FAH	1.2	1-1.3	0.00698	0.94	0.75-1.17	0.58805
2137	EXTL3	1.2	1.1-1.4	0.005	1.19	0.94-1.51	0.14242
8706	B3GALNT1	1.2	1-1.3	0.00809	1.01	0.86-1.19	0.88426
7378	UPP1	1.2	1.1-1.3	0.00014	1.09	0.95-1.24	0.21579
4835	NQO2	1.2	1-1.4	0.00777	0.92	0.77-1.09	0.33263
11253	MAN1B1	1.2	1.1-1.5	0.009	0.99	0.77-1.27	0.93416
1261	CNGA3	1.1	1-1.2	0.00299	1.03	0.94-1.12	0.51853
9488	PIGB	1.3	1.1-1.4	9.00E-05	1.04	0.86-1.27	0.66946
3073	HEXA	1.3	1.1-1.5	0.00113	1.07	0.84-1.36	0.56997
1534	CYB561	1.3	1.1-1.4	0.00153	1.21	0.96-1.52	0.10342
35	ACADS	1.6	1.2-2.2	0.00175	1.18	0.81-1.73	0.38462
10577	NPC2	1.2	1-1.3	0.00705	0.92	0.73-1.15	0.45549
10723	SLC12A7	1.2	1.1-1.4	0.00167	0.98	0.81-1.19	0.85272
8309	ACOX2	1.2	1.1-1.3	0.00082	1.03	0.9-1.18	0.6778
11285	B4GALT7	1.4	1.1-1.8	0.00496	1.02	0.73-1.43	0.90036

**Table 2:** Clinical characteristics of GBM patients from the TCGA, GEO (GSE4271, GSE4412 and GSE16011) datasets.

Characteristics	TCGA	GSE4271	GSE4412	GSE16011
Sample No.	529	77	85	276
Gender*-M/F	311/202	51/25	32/53	184/92
Age (year)*^a^	58.7(10.9-86.6)	48(22-82)	42(18-82)	51.5(11.7-81.2)
Chemotherapy*-yes/no	350/89			
Radiotherapy*-yes/no	434/64			
Tumor diameter (cm)*^a^	1(0.3-3.0)			
Survival status*-dead/alive	422/89	15/62	59/26	237/35
Overall survival time (days)*	370(3-3881)	665(21-3339)	389(7-2516)	452(0-7548)

* Data is missing. ^a^ Age, Tumor diameter and Overall survival time were expressed as median (range)

## Abbreviations

TCGA: The Cancer Genome Atlas
KEGG: Kyoto Encyclopedia of Genes and Genomes
GSEA: Gene Set Enrichment Analysis
WHO: World Health Organization
MGMT: Methylation of O-6-methylguanine-DNA methyltransferase
UCSC: University of California Santa Cruz
GEO: Gene Expression Omnibus
AUROC: area under receiving operating characteristic
PFS: progression-free survival
