# Supplementary material for: Twenty Metabolic Genes Based Signature Predicts Survival of Glioma Patients
Source: J Cancer. 2020 Jan 1;11(2):441–9. doi: 10.7150/jca.30923 (PMC6930419; doi:10.7150/jca.30923)
Supplement: Supplementary file 1 — Supplementary figures and tables. [file jcav11p0441s1.pdf]

Table S1. Function of 20 candidate genes.

Gene Symbol	Gene Function	Reference
ALOX5AP	required for leukotriene synthesis with 5-lipoxygenase	RefSeq, Feb 2011
ACAA2	catalyzing the last step of the mitochondrial fatty acid beta-oxidation spiral, **providing a possible linkage between fatty acid metabolism and apoptosis of cells**	RefSeq, Jul 2008; PMID: 18371312
PYGL	homodimeric protein, catalyzing the cleavage of alpha-1,4-glucosidic bonds to release glucose-1-phosphate from liver glycogen stores	RefSeq, Feb 2011
OAS1	synthesizing 2',5'-oligoadenylates (2-5As), **associated with Lipopolysaccharide-induced cell death**	RefSeq, Feb 2016; PMID: 26516113
NNMT	responsible for N-methylation, **associated with pancreatic cancer, bladder cancer, ovarian cancer, colorectal cancer, clear cell Renal cell carcinoma**	RefSeq, Jul 2008; PMID: 29974846; PMID: 29148015; PMID: 27323852; PMID: 26942567; PMID: 28412735
FAH	the last enzyme in the tyrosine catabolism pathway	RefSeq, Jul 2008
EXTL3	a glycosyltransferase, catalyzing the transfer of N-acetylglucosamine to glycosaminoglycan chains,	RefSeq, Nov 2012; PMID: 28132690

	**associated with neurological abnormality**	
B3GALNT1	beta-1,3-galactosyltransferase, with diverse enzymatic functions, **a potential therapy target gene to suppress metastasis in non-small cell lung cancer**	RefSeq, Mar 2017; PMID: 25521548
UPP1	uridine phosphorylase, functioning in the degradation and salvage of pyrimidine ribonucleosides, **may be an independent prognostic marker in breast carcinoma**	RefSeq, Oct 2016; PMID: 11807789
NQO2	catalyzing the two-electron reduction of quinone substrates, **associated with neurodegenerative diseases and several cancers**	RefSeq, Mar 2014; PMID: 26609153; PMID: 21803122; PMID: 21351093
MAN1B1	functioning in N-glycan biosynthesis, **associated with poor prognosis and modulating proliferation and apoptosis in bladder cancer**	RefSeq, Dec 2011; PMID: 30218751
CNGA3	required for normal vision and olfactory signal transduction	RefSeq, Jul 2008
PIGB	located in the endoplasmic reticulum and involved in GPI-anchor biosynthesis	RefSeq, Jul 2008
HEXA	catalyzing molecules containing terminal N-acetyl hexosamines, **associated with prostate cancer**	RefSeq, Jan 2016; PMID: 24389457
CYB561	a senescene-associated gene in normal human oral keratinocytes, associated with the adrenergic pathway	PMID: 24140660; PMID: 12837283

ACADS	catalyzing the initial step of the mitochondrial fatty acid beta-oxidation pathway	RefSeq, Oct 2014
NPC2	functioning in regulating the transport of cholesterol, **associated with Niemann-Pick disease, type C2 and frontal lobe atrophy**	RefSeq, Jul 2008; PMID: 27792009
SLC12A7	**associated with adrenocortical carcinoma tumorigenesis and gynecological cancers**	PMID: 26454676; PMID: 15262997
ACOX2	involved in the degradation of long branched fatty acids and bile acid intermediates in peroxisomes, **a potential novel therapeutic biomarker in ER+ breast tumors**	RefSeq, Mar 2009; PMID: 26183823
B4GALT7	beta-1,4-galactosyltransferase, associated with the progeroid form of Ehlers-Danlos syndrome	RefSeq, Oct 2009; PMID: 20809901

The gene functions marked in bold in the table show the possibility that the gene is associated with glioma.
